# Fibroblast-derived matrix models desmoplastic properties and forms a prognostic signature in cancer progression

**DOI:** 10.3389/fimmu.2023.1154528

**Published:** 2023-07-18

**Authors:** Maria Rafaeva, Adina R. D. Jensen, Edward R. Horton, Kamilla W. Zornhagen, Jan E. Strøbech, Lutz Fleischhauer, Alejandro E. Mayorca-Guiliani, Sebastian R. Nielsen, Dina S. Grønseth, Filip Kuś, Erwin M. Schoof, Luis Arnes, Manuel Koch, Hauke Clausen-Schaumann, Valerio Izzi, Raphael Reuten, Janine T. Erler

**Affiliations:** ^1^Biotech Research and Innovation Centre, University of Copenhagen, Copenhagen, Denmark; ^2^Center for Applied Tissue Engineering and Regenerative Medicine-CANTER, Munich University of Applied Sciences, Munich, Germany; ^3^Center for NanoScience – CsNS, Ludwig-Maximilians-University Munich, Munich, Germany; ^4^Department of Biotechnology and Biomedicine, Technical University of Denmark, Lyngby, Denmark; ^5^The Finsen Laboratory, Rigshospitalet, Faculty of Health Sciences, University of Copenhagen, Copenhagen, Denmark; ^6^Novo Nordisk Foundation Centre for Stem Cell Biology, DanStem, Faculty of Health Sciences, University of Copenhagen, Copenhagen, Denmark; ^7^Center for Biochemistry, Center for Molecular Medicine Cologne (CMMC), Faculty of Medicine and University Hospital Cologne, University of Cologne, Cologne, Germany; ^8^Institute for Dental Research and Oral Musculoskeletal Biology, Faculty of Medicine and University Hospital Cologne, University of Cologne, Cologne, Germany; ^9^Faculty of Biochemistry and Molecular Medicine, University of Oulu, Oulu, Finland; ^10^Faculty of Medicine, University of Oulu, Oulu, Finland; ^11^Foundation for the Finnish Cancer Institute, Helsinki, Finland; ^12^Institute of Experimental and Clinical Pharmacology and Toxicology, Medical Faculty, University of Freiburg, Freiburg, Germany; ^13^Department of Obstetrics and Gynecology, Medical Center, University of Freiburg, Freiburg, Germany

**Keywords:** extracellular matrix, fibroblasts, mechanics, models, desmoplasia, pancreatic cancer, breast cancer

## Abstract

The desmoplastic reaction observed in many cancers is a hallmark of disease progression and prognosis, particularly in breast and pancreatic cancer. Stromal-derived extracellular matrix (ECM) is significantly altered in desmoplasia, and as such plays a critical role in driving cancer progression. Using fibroblast-derived matrices (FDMs), we show that cancer cells have increased growth on cancer associated FDMs, when compared to FDMs derived from non-malignant tissue (normal) fibroblasts. We assess the changes in ECM characteristics from normal to cancer-associated stroma at the primary tumor site. Compositional, structural, and mechanical analyses reveal significant differences, with an increase in abundance of core ECM proteins, coupled with an increase in stiffness and density in cancer-associated FDMs. From compositional changes of FDM, we derived a 36-ECM protein signature, which we show matches in large part with the changes in pancreatic ductal adenocarcinoma (PDAC) tumor and metastases progression. Additionally, this signature also matches at the transcriptomic level in multiple cancer types in patients, prognostic of their survival. Together, our results show relevance of FDMs for cancer modelling and identification of desmoplastic ECM components for further mechanistic studies.

## 1 Introduction

The extracellular matrix (ECM) is a protein scaffold to which cells adhere, that provides both biochemical and biophysical cues in order to maintain organ homeostasis and integrity (1). The ECM reveals a vital impact on cancer progression, as cancer cells invade the ECM at the primary tumor and further interact with ECM proteins at different stages of metastasis (2). Secondary to physiological insults, such as wounds, a desmoplastic reaction regularly occurs in cancer (3). Several studies have shown that increased inflammation is a precursor of cancer (4), and tumors are often described as “wounds that do not heal” (5). Such reaction of the cancerous tissue stroma results in overproduction and deposition of ECM concomitant with increased proliferation of myofibroblasts in the tumor microenvironment (TME) and lacks regaining of normal tissue homeostasis (6).

This desmoplastic reaction is a defining feature of breast- and pancreatic cancers and is correlated with poor prognosis (7, 8). In pancreatic adenocarcinoma (PDAC), desmoplasia has largely been attributed to the physical properties of the tumor stroma, that impedes drug delivery, response to radiation, and increases metastasis to other organs, primarily the liver (9). At the primary site in breast cancer, there is also an excessive deposition of the ECM, mainly collagen I, and remodeling, which leads to linearization of the fibers forming ‘tracks’ for cell migration from the tumor margin (10). This ECM reorganization is usually present at the invasive stage and is used prognostically, with increased observation in the stroma correlating with poor outcome (11, 12). Linearization of the ECM emerges from the cross-linking of fibers with enzymes, primarily lysyl-oxidases (LOX, LOXL1-4) and the physical compacting of the ECM by dividing cells (13) resulting in a denser and stiffer ECM (14). In turn, stiffened matrix boosts β1-integrin activity and thereby focal adhesion formation of the stromal cells in the TME, transforming mechanotransduction into pro-tumorigenic cell signaling responses (15, 16). For instance, transcriptional activation of Yes-associated protein (YAP) with increasing ECM stiffness promotes the proliferation and migration of breast cancer cells upon transduction of signals from the focal adhesions (17, 18). In addition, integrin-independent mechanotransduction was shown to activate the EPHA2/LYN kinase that promotes epithelial to mesenchymal transition (EMT) and subsequently tumor cell invasion in breast cancer (19).

More than 80% of the ECM is produced by stromal cells (20). Cancer-associated fibroblasts (CAFs) are key contributors to the deposition and remodeling of the ECM during cancer progression, in both breast and pancreatic cancer (21, 22). Normal fibroblasts (NFs) and CAFs are commonly used in studies to better understand cancer progression, especially with respect to cancerous transformation and cell-cell communication in the TME (23, 24). However, their contribution to the composition and organization of the ECM and influence of this on cancer cell proliferation has so far not been investigated in detail.

Understanding the specific role of ECM characteristics during cancer progression is becoming increasingly important in order to improve drug efficacy and identify potential therapeutic targets for better patient outcomes. To study the influence of the fibroblast-derived ECM, we utilized fibroblast-derived matrices (FDMs) (25) that resemble the ECM composition detected in decellularized mouse organs (26). Here, we show that although cancer cells equally adhere to the normal and cancerous matrices, they show increased proliferation on CAF FDMs. The CAF FDM composition mimics core changes in desmoplastic metastatic cancer *in vivo*. Strikingly, CAFs assemble denser and stiffer ECM than NFs, structurally resembling tumor matrix. We derive a 36-gene matrisome signature based on CAF ECM, which shows enrichment in multiple cancer types in humans and is prognostic of several cancer types’ outcome.

## 2 Materials and methods

### 2.1 Cell lines

The 4T1 mouse mammary carcinoma cell line was a kind gift of Fred Miller (Wayne State University). The KPCmT4 murine pancreatic cancer cell line was isolated from PDAC tumor tissue obtained from KrasLSL-G12D/+; Trp53LSL-R172H/+; Pdx1–Cre mice of a pure C57BL/6 background and were gifted by the Tuveson laboratory (Cold Spring Harbor Laboratory, NY, USA) (27). Cell lines were cultured in Dulbecco’s modified Eagle medium GlutaMAX (DMEMGlutaMAX; Gibco, Thermo Fisher Scientific, cat. no. 10566016, Grand Island, NY, USA) supplemented with 1% penicillin-streptomycin (PS, 100 U/mL, Gibco, Thermo Fisher Scientific) and 10% fetal bovine serum (FBS, Gibco, Thermo Fisher Scientific). The 4T1-H2B-GFP+ cells were previously generated by stable transfection of 4T1 cells with a pBOS-H2BGFP vector (BD Pharmingen, San Jose, CA, USA) (28). The KPCmT4-zsGreen cells were generated by stable transfection of KPCmT4 cells with pHIV Luc-zsGreen vector (gift from B. Welm, University of Utah, USA, Addgene plasmid no. 39196). Immortalized mCAF1 and mNF1 murine fibroblast cell lines were a kind gift of Erik Sahai (The Francis Crick Institute, London UK), isolated from the mammary tumor, and healthy fat pad of FVB/n MMTV-PyMT mouse line, respectively (23). Cells were cultured in DMEM high glucose (Gibco, Thermo Fisher Scientific) with 10% FBS, 1% Insulin Transferrin Selenium Solution (ITS-G Gibco, Thermo Fisher Scientific) and 1% PS. All cell lines were regularly tested for mycoplasma and maintained at a 37°C, 5% CO2 humidified atmosphere.

### 2.2 Fibroblast-derived matrices

Fibroblast-derived matrices (FDMs) from the mCAF1 and mNF1 cell lines were generated as previously described (25). Briefly, fibroblasts were seeded on cross-linked gelatin dishes, and treated for 7 days with 50µg/ml ascorbic acid daily. Afterwards, fibroblasts were treated with 20 mM NH_4_OH with 0.5% Triton-X-100 for 5 minutes, followed by a gentle wash in PBS. 0.5% sodium deoxycholate (Sigma-Aldrich, D6750-500 mg) was then added for 60 minutes at room temperature, and then removed. The FDMs were washed in PBS and then DNase I (10 μg/ml in PBS) was added for 60 minutes at 37 °C. This protocol was optimized for either a 6-well plate (volumes 1 ml/well), 12- and 24- well plate (volumes 0.5 ml and 0.25 ml/well) or a 96-well plate (volumes 0.1 ml/well). If not immediately used, they were stored at 4°C in PBS supplemented with 1% PS. For optimizing cell-derived matrix generation and use, reader can further consult with previously published protocols (29–31).

### 2.3 KPC mouse samples generation

KPC mice (Tg(Pdx1-cre)6TuvKrastm4TyjTrp53tm2Tyj) were imported from The Beatson Institute for Cancer Research (Glasgow, UK) and originally established from Jax stocks #014647, #008180, #008652 in a mixed background (32). Three mice (both sexes) per group were used. As control groups, we used age-matched Pdx1-cre+ mice. KPC mice were used at pancreatic intraepithelial neoplasia (PanIN) stage (3-4 months old), early PDAC tumor stage (4.5 months old) and late tumor stage (5-8 months old). For generating decellularized tissue, pancreas/pancreatic tumors and livers in the same animal were perfused according to previously published protocol (33). For the liver metastases group were selected mice with developed tumor where was observed macroscopic metastasis, resection of area from decellularized livers was performed based on 2 knots of 9-0 suture marking prior decellularization. After perfusion and washes in MQ water samples were resected and snap frozen for further storage at -80°C.

### 2.4 Intrasplenic KPC injections and sample generation

Female C57BL/6 mice (6–12 weeks old; Taconic, Denmark) were used for intrasplenic injections of KPCmT4 cells at 1 million cells per 50µL of PBS (34). Healthy matched mice were used as a control, 3 mice per group. 20 days post-injection livers were decellularized according to (33) and samples were resected and snap frozen after perfusion washes with MQ water.

### 2.5 Decellularized tissues for mass spectrometry sample preparation

Decellularized tissue samples were defrosted, and tissues were punched under dissection microscope (Greenough, with two-armed gooseneck; Leica, model no. S6 D) with 2mm punch biopsy tools (Harris Uni-Core) and weighed (tools thoroughly cleaned between samples with methanol). Lysate preparation and digestion was done according to (35) with modifications. Briefly, ~6mg of decellularized tissue pieces were lysed using 30 µl of lysis buffer (consisting of 6 M Guanidinium Hydrochloride, 10 mM TCEP, 40 mM CAA, 50 mM HEPES pH 8.5) in Barocycler 2320EXT (Pressure BioSciences) set to 30 cycles of 45,000 p.s.i., 50 seconds on, 10 seconds off. Samples were boiled at 95 °C for 5 minutes, after which they were sonicated on the ‘high’ setting for 5 × 30 seconds in a Bioruptor sonication water bath (Diagenode) at 4°C. KPC intrasplenic samples were filtered through Microcon centrifugal unit with 30kDa cut-off (cat. no. Z648086, Millipore). After determining protein concentration with Bradford reagent (cat. no. B6916, Sigma), 20 µg was taken forward for digestion.

Samples were diluted 1:3 with 10% Acetonitrile, 50 mM HEPES pH 8.5, LysC (MS grade, Wako) was added in a 1:50 (enzyme to protein) ratio, and samples were incubated at 37°C for 4 hours. Samples were further diluted to 1:10 with 10% Acetonitrile, 50 mM HEPES pH 8.5, trypsin (MS grade, Promega) was added in a 1:100 (enzyme to protein) ratio and samples were incubated overnight at 37°C. Enzyme activity was quenched by adding 2% trifluoroacetic acid (TFA) to a final concentration of 1%. Prior to mass spectrometry analysis, the peptides were desalted on in-house packed C18 Stage tips. For each sample, 2 discs of C18 material (3M Empore) were packed in a 200µl tip, and the C18 material activated with 40µl of 100% Methanol (HPLC grade, Sigma), then 40µl of 80% Acetonitrile, 0.1% formic acid. The tips were subsequently equilibrated 2 x with 40µl of 1%TFA, 3% Acetonitrile, after which the samples were loaded using centrifugation at 4,000 x rpm. After washing the tips twice with 100µl of 0.1% formic acid, the peptides were eluted into clean 500µl Eppendorf tubes using 40% Acetonitrile, 0.1% formic acid. The eluted peptides were concentrated in an Eppendorf Speedvac, and reconstituted in 1% TFA, 2% Acetonitrile for Mass Spectrometry (MS) analysis.

### 2.6 FDM mass spectrometry sample preparation

Lysates of the mCAF1 and mNF1 FDMs were collected in biological triplicates. All lysates were washed in 1 x PBS, scraped, and collected. Samples were centrifuged at 8000g for 10 minutes at 4°C. PBS removed, and 20 µL lysis buffer added (6 M Guanidinium Hydrochloride, 10 mM TCEP, 40mM CAA, 100 mM Tris pH8.5). Samples were vortexed and boiled for 5 minutes at 95°C for 5 minutes. Samples were then sonicated using the Bioruptor 5 x 30 seconds on/30 seconds off using maximum setting. Samples were then centrifuged 1 min, 13,000 rpm and snap frozen in liquid nitrogen. Sample preparation and acquisition were then performed as previously described (26).

### 2.7 Mass spectrometry acquisition and analysis

#### 2.7.1 KPC samples

For each sample, peptides were loaded onto a 2cm C18 trap column (cat. no.164705, Thermo Fisher), connected in-line to a 75 cm C18 reverse-phase analytical column (cat. no. ES805, Thermo EasySpray) using 100% Buffer A (0.1% Formic acid in water) at 750bar, using the Thermo EasyLC 1000 HPLC system, and the column oven operating at 45°C. Peptides were eluted over a 200-minute gradient ranging from 6 to 60% of 80% acetonitrile, 0.1% formic acid at 250 nL/minute, and the Q-Exactive instrument (Thermo Fisher Scientific) was run in a DD-MS2 top10 method. Full MS spectra were collected at a resolution of 70,000, with an AGC target of 3×10^6^ or maximum injection time of 20 milliseconds and a scan range of 300–1750 m/z. The MS2 spectra were obtained at a resolution of 17,500, with an AGC target value of 1×10^6^ or maximum injection time of 60 milliseconds, a normalized collision energy of 25 and an intensity threshold of 1.7 ×10^4^. Dynamic exclusion was set to 60 seconds, and ions with a charge state < 2 or unknown were excluded. MS performance was verified for consistency by running complex cell lysate quality control standards, and chromatography was monitored to check for reproducibility.

#### 2.7.2 Analysis of all samples

The raw files were analyzed using Proteome Discoverer 2.4. Label-free quantitation (LFQ) was enabled in the processing and consensus steps, and spectra were matched against the Mus Musculus database obtained from Uniprot. Dynamic modifications were set as Oxidation (M), Deamidation (N, Q) and Acetyl on protein N-termini. Cysteine carbamidomethyl was set as a static modification. All results were filtered to a 1% FDR, and protein quantitation done using the built-in Minora Feature Detector. Normalization was performed in the total peptide amount mode, which sums the peptide group abundances for each sample and determines the maximum for all files, then using it as a normalization factor. At post-processing of the dataset proteins were sorted for identified based on 2 or more unique peptides in addition to be quantified among three biological repeats. Statistical analysis was performed using Limma package of R studio software. LogFC values were calculated as a difference of the means. A linear model was fit to the data, following an empirical Bayes moderated t-test and p-values adjustment for multiple testing with Benjamini-Hochberg method. Proteins were next sorted for ‘in silico’ defined matrisome (36). For heatmaps generation were used Cluster 3.0 (C Clustering Library 1.59) and visualization was done using Java Tree View (version 1.2.0).

### 2.8 Second harmonic generation (SHG) imaging of FDMs combined with fluorescence imaging

For imaging FDMs have been deposited as described on glass bottom 24 or 12-well plates (cat. no. P24-1.0-13-F, MatTek) with fibroblast cell number and volumes adjusted to the area of the wells. After staining, FDMs were stored in 1% PS/PBS at +4°C. FDMs were imaged on the inverted Leica SP5-X confocal microscope with a two-photon laser (Spectra-physics, Mai Tai DeepSee model; range 680-1,040nm) adjusted to 880nm and SHG was detected by hybrid detector (at 420-460, Leica, HyD S model). Alexa-488 secondaries were detected simultaneously by PMTs (Leica). We used two different objectives for imaging - lambda blue, 20×, 0.70 numerical aperture (NA) IMM UV; Leica, HCX PL APO model and 40x, 1.3 NA OIL UV; Leica, HCX PL APO CS. SHG imaging stacks were acquired at 512x512 pixels, 100Hz, 1 line averaging with a 2.5um z-step using 40x objective. Antibody staining was acquired at 1024x1024 pixels, 100Hz, 1 line averaging with 2.5µm z-step using 20x objective. For data acquisition, Leica Application Suite (LAS) version 4 microscope software was used.

### 2.9 Analysis of FDM density and thickness

For analysis of fibrillar collagens density, single planes with the largest presence of the ECM were selected from SHG z-stacks (40x acquired). Brightness of images was equally adjusted, and images were processed in Fiji software with Twombli plugin to measure high density matrix (37). For analysis of FDM thickness, SHG signal was measured across planes of z-stacks (40x acquired) in Fiji software. Number of planes with signal were counted and multiplied by z-step size in order to estimate thickness in µm.

### 2.10 Indentation-type atomic force microscopy

mNF1 and mCAF1 FDMs were produced as described above in 35 x 10 mm petri dishes (cat. no. 353001, FALCON). Stiffness measurements were carried out using a NanoWizard I AFM (JPK BioAFM Bruker Nano GmbH, Berlin, Germany) in combination with an inverted optical microscope (Axiovert 200, Carl Zeiss Micro Imaging GmbH, Göttingen, Germany). To avoid external disturbance during measurement, the whole setup is placed on an active vibration isolation table (Micro 60, Halcyonics, Göttingen, Germany) inside a self-build 1 m³ soundproof box. The AFM was used in the indentation mode with pyramidal shaped tips with a radius of around 20 nm and a spring constant of 0.1 N/m. For each cantilever the spring constant and the sensitivity were determined individually using the thermal noise method (38). During measurements, the matrix was immersed in PBS (Biochrom Dulbecco’s PBS w/o Mg2+/Ca2+, pH 7.4, Berlin, Germany). On each obtained matrix 6, force maps of 5 x 5 indentation curves equally distributed in an area of 30 x 30 µm were obtained. Indentations were made up to 1.5 V with a speed of 10 µm/s and calibration was performed after the experiment. The six force map locations were arbitrary chosen. During measurements the cantilever was retracted in vertical direction (z-axis) up to 50 µm and therefore the CellHesion^®^ module (JPK BioAFM Bruker Nano GmbH, Berlin, Germany) was used. The Young’s Modulus was extracted by fitting the Hertz-Sneddon model for a pyramidal indenter to the whole approach part of the force-indentation curves, using the JPK Data Processing Software (Version 5.0.96, JPK Instruments).

### 2.11 FDM staining with antibodies and CNA35 probe

Stored FDMs in 1% PS/PBS at 4°C were brought to room temperature (RT). Next, FDMs were gently washed with PBS following blocking in 3% donkey serum (cat. no.017-000-121, Jackson ImmunoResearch), 1% BSA/PBS solution for 1 hour at RT. After, matrices were gently washed with PBS and covered with primary antibody dilution [1% BSA/PBST (0,05% Tween)]. Primary antibody used: rabbit anti-periostin (polyclonal KR131, provided by M. Koch), rabbit anti-collagen XII (polyclonal KR145, provided by M. Koch), rabbit anti-collagen VIα1C (polyclonal, provided by R. Wagener) at 1:100 dilution. After overnight incubation at 4°C, matrices were gently washed 3x5 minute in PBS-0.2% Tween and then secondary antibody solution has been applied - 1% BSA/PBST with 1:500 donkey anti-rabbit AlexaFluor488 IgG (H+L) (A-21206, Thermo Fisher Scientific) for 1 hour at RT. Finally, samples were gently washed 3x5 min in PBST and stored in 1% PS/PBS at 4°C until imaging.

FDMs were also stained with in-house produced anti-collagen CNA35-mCherry probe. The probe was produced according to (39) with a few modifications. Briefly, streaked bacterial colony of BL21(DE3) strain carrying a plasmid pET28a-mCherry-CNA35 (Addgene #61607) was inoculated in 20ml kanamycin (50µg/ml) containing LB medium (4529, SSI Diagnostica) and next day further expanded during overnight culture in 2L NZY Auto-Induction LB medium (1/100) (MB179, NZYtech). After centrifugation, bacterial pellet was lysed in NZY Bacterial Cell Lysis Buffer supplemented with Lysozyme (50mg/ml) and DNase I (2mg/ml) and frozen at -20°C until protein extraction. Cleared from residual cell debris lysate was diluted with 0.5M NaH2PO4 pH 7,6 (1:10) and applied on a washed and equilibrated pre-elution gelatin-sepharose (17-0956-01, GE Healthcare) column connected to elution column with PureCube 100 INDIGO Ni-Agarose (75105, Cube Biotech). After two washes with 10mM Tris, 150mM NaCl (pH = 7.6), elution was performed by loading sequentially 5,10,20,30,60,80,150, 300 mM Imidazol in 20mM Tris, 200mM NaCl (pH = 7.6) solutions. Three last fractions were collected and dialysis of those was performed against 1 x PBS. Protein concentrations were measured in all fractions, probe was protected from light and sterile filtered (Ultrafree-cl gv 0.22um sterile (UFC40GV0S, Millipore) prior being aliquoted and stored at -20°C. For staining, FDMs were incubated with 1µM CNA35-mCherry in PBS at RT overnight and washed with PBS before imaging.

### 2.12 Cell adhesion and proliferation

**Figure 1:**
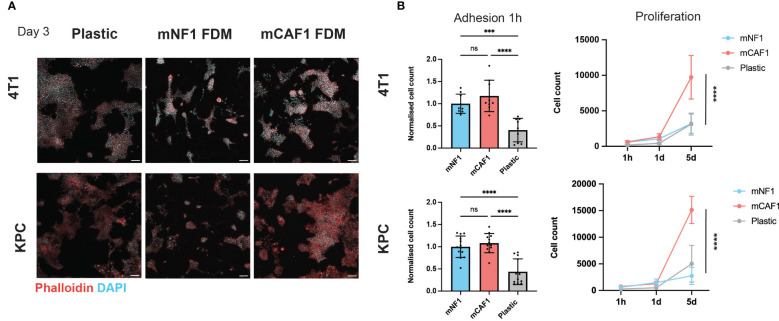
CAF FDM promotes breast and pancreatic cancer cells proliferation. **(A)** Representative images of 4T1-H2B-GFP breast cancer and KPCmT4-zsGreen pancreatic cancer cells on plastic, mNF1, and mCAF1 FDMs. Day 3 post-seeding in 2% FBS DMEM. Staining with phalloidin (F-actin) and DAPI (nuclei). **(B)** Adhesion (1 hour) and proliferation (1-5 days) of 4T1-H2B-GFP and KPCmT4-zsGreen cells seeded on plastic, mNF1, mCAF1 FDMs. Normalized or raw cell count based on DAPI and GFP. n=3 repeats. One-way ANOVA test. ns, p-value > 0.05; ***p-value < 0.001, ****p-value < 0.0001. Scale bars 100 μm.

Cell adhesion and proliferation assays were performed using 4T1-H2B-GFP and KPCmT4-zsGreen cells. 5000 (in 2% serum DMEM) cells were seeded in high content 96-well imaging plates (Corning, 3340) on wells containing mCAF1 FDMs, mNF1 FDMs or on plastic. Cells were allowed to attach for 1 hour, after which plates for adhesion were fixed in 10% formalin (Formalin solution 10% neutral buffered, Sigma-Aldrich, cat. no. HT501128-4L) (100uL per well) for 10 minutes. To follow proliferation, additional plates were fixed at 1 day and 5 days post-seeding. Cells were permeabilized in 0.2% Triton-X-100 in PBS (Sigma-Aldrich, cat. no. T8787-50 mL) for 2–5 minutes. Next 2×PBS washes were performed, after which DAPI was added at 1 μg/mL for 90 min at room temperature. Plates were then washed 3x5 minutes in PBS, placed in 100 μL PBS and stored at 4°C in the dark until imaging. Imaging was performed on the INCell Analyzer 2200 (GE Healthcare Life Sciences). Images were analyzed using the INCell Analyzer Workstation 1000 software (GE Healthcare Life Sciences). Nuclei were segmented based on the DAPI staining using the Tophat segmentation method. The mean intensity of GFP and DAPI in each nucleus were measured, and the number of GFP positive and DAPI positive cells were counted and compared between conditions. Note: Images in **Figure 1A**. are acquired from a 24-well plate (cat. no. P24 -1.0-13F, MatTek Corporation) where cell seeding densities were adjusted to the area of the wells and wells were equilibrated with cell culture medium prior seeding. Samples were prepared, fixed and permeabilized as described above after 3 days, stained with DAPI (1 μg/mL) to visualize cell nuclei and AlexaFluor633-phalloidin (1:500; A22284, Thermo Fisher Scientific) to visualize cell bodies for 1 hour at room temperature. Imaging was performed on Leica SP8 confocal microscope with HC Plan-Apochromat 10x/0.40 AI at 1024x1024 pixels resolution, 5 μm z-step.

mNF1 and mCAF1 fibroblasts proliferation was assessed in the following way. 2000 cells were seeded in 96-well plates and cultured under regular conditions for three days (72h). Afterwards, triplicates and background wells measurements were performed 1 hour after incubation with CellTiter 96 Aqueous One Solution Cell Proliferation Assay (Promega, G3580) according to the manufacturers instructions.

### 2.13 Western blot validation of mNF1/mCAF1 FDMs

FDMs were lysed in 9M Urea with 1% 2-Mercaptoethanol. Lysates were shaken rigorously for 45 minutes at 4°C before 5 minutes of sonication (30 sec. on 30 sec. off). Lysates were boiled for 5 minutes before centrifugation at 15,000 rpm for 15 minutes at 4°C. Protein lysates were resolved on NuPAGE 4 –12% Bis-Tris gels (Thermo Fisher Scientific, cat. no.17080971) and transferred to nitrocellulose membranes. Membrane was stained with Ponceau stain (**Supplementary Materials**) (Sigma-Aldrich, cat. no. P7170). Membranes were blocked in 5% milk for 1 hour and incubated with primary antibodies overnight at 4°C. Primary antibodies included LOX, Cell Signaling D8F2K (1:1000), Collagen IV Sigma-Aldrich AB756P (1:1000), Collagen XII KR144 (1:1000; provided by M. Koch). The next day, membranes were washed with TBS-Tween and incubated with appropriate HRP-conjugated secondary antibodies for one hour. Immunoblots were visualized on an ImageQuantTM LAS 400 instrument and images were analyzed using ImageJ.

### 2.14 Desmoplastic signature analysis from human dataset

The long signature used for this study comprises of the following 36 proteins: Col1a1, Col1a2, Col5a2, Col6a1, Col6a2, Col8a1, Col12a1, Col14a1, Col16a1, Dpt, Emilin1, Fn1, Fbn1 Mfap2, Mfap5, Postn, Tgfbi, Thbs1, Thbs2, Thsd4, Tnc, Tsku, Vwa1, Aspn, Adamts2, Bmp1, Cd109, Lox, Loxl2, Mmp19, Serpine1, Timp1, Angptl4 and Crlf1. The short signature comprises 9 of these proteins: Col1a2, Col5a2, Col12a1, Fn1, Mfap5, Postn, Tgfbi, Thbs2, Lox. These proteins were selected based on, LogFC > 1.5, p-value < 0.05).

The entire database from the Celligner/DepMap tool (40) was locally downloaded and the single-sample gene set enrichment value of the desmoplastic fibroblast signature evaluated across 10070 patients from primary tumors of 29 tissues (67 tumor subtypes) using the singscore package (41) in R. Tumor-wise differences were evaluated using one-way ANOVA, followed by Tukey HSD test. ANOVA p-value for all comparisons was p < 1*10^-16^. Matrisome genes were defined in (42) and downloaded from the Matrisome Project portal at http://matrisome.org/. In each test, the entire cohort was scored, and the results presented. Additionally, single genes were scored individually in the same way, results of which are shown in **Supplementary Figure 8**.

### 2.15 Analysis and statistics

Statistical analyses other than proteomics datasets were performed in Prism 9 (version 9.4.1). All data was tested for normal distribution, following which the appropriate statistical analysis was performed. Significance was p-value < 0.05 throughout, apart from mass spectrometry data, where it defined as p-value < 0.1 for *in vivo* dataset. Statistical analyses were performed using unpaired t-test, or analysis of variance (ANOVA, where there were multiple comparison groups).

## 3 Results

### 3.1 CAF FDM stimulates cancer cells proliferation

For this study, we selected NF (mNF1) and CAF (mCAF1) fibroblasts generated from FVB and PyMT-FVB mice, respectively, which are present in the normal fat pad and late tumor stage. These immortalized and well-characterized cells (23) allowed the generation of sufficient FDMs in a span of 7 days, which is the shortest timeframe described for FDM deposition (25, 29). We probed cancer cell response, adhesion and proliferation, on these matrices versus regular tissue culture plastic (**Figure 1A**). We chose two cell lines; triple-negative breast cancer cells (4T1) and pancreatic cancer cells (KPCmT4), given these are highly desmoplastic diseases. Firstly, we assessed cell number of the cancer cells upon adhesion to the FDMs and plastic (1 hour post-seeding), which showed no difference between NF and CAF FDMs (**Figure 1B**). Quantification of the cells at two additional time points (1 day, 5 days) showed that both cell types proliferate more on the CAF FDM comparing to NF. Hence, CAF FDM might instruct the cancer cell proliferative potential.

### 3.2 CAF FDM structure reflects *in vivo* desmoplastic ECM

**Figure 2:**
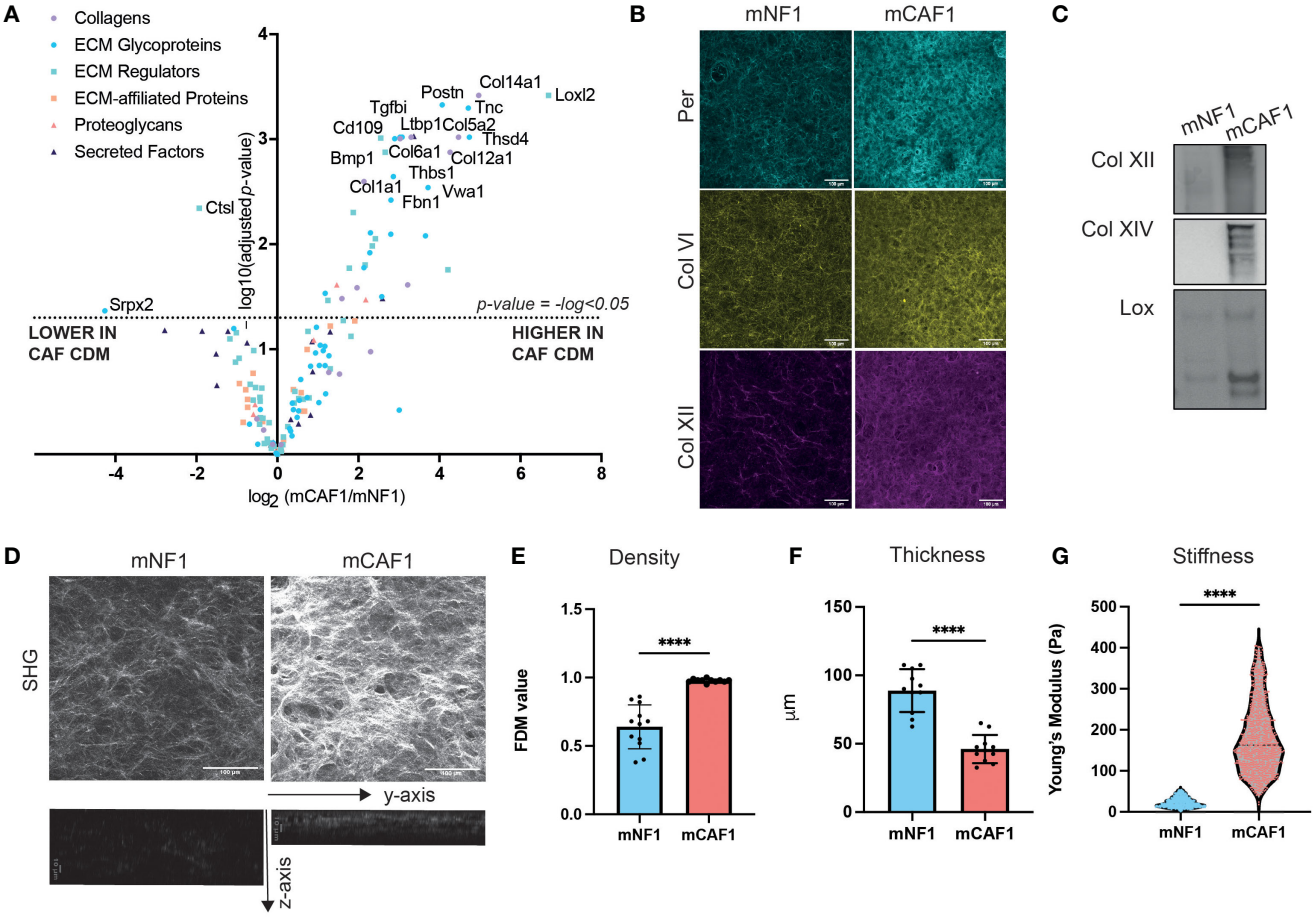
CAF FDM is compositionally and structurally different from NF FDM. **(A)** Volcano plot showing ECM composition difference between mCAF1 vs mNF1 FDMs. n= 3 samples per condition. **(B)** Validation of mCAF1 vs mNF1 upregulated proteins (Per - periostin, ColIV - collagen IV, Col XII - -collagen XII) by immunofluorescent staining of FDMs. **(C)** Validation of mCAF1 vs mNF1 upregulated proteins (ColXII, ColXIV - collagen XIV, LOX - lysyl oxidase) by western blotting.**(D)** Representative images of mNF1 and mCAF1 FDMs imaged by second harmonic generation (SHG). Maximum intensity projection and y-z projection. **(E)** mNF1 and mCAF1 FDMs density (based on single plane analysis). n= 3 repeats. **(F)** mNF1 and mCAF1 FDMs thickness (based in y-z projection analysis). n=3 repeats. **(G)** mNF1 and mCAF1 FDMs stiffness based on the atomic force microscopy measurements. n = 5-7 matrices. Unpaired t-test- ****p-value <0.0001. Scale bars 100 um, except z-axis images with 10um scale bar.

In order to investigate whether changes in composition of the cancerous ECM were causing the alterations in proliferation observed, we next characterized the composition of both FDMs by label-free mass spectrometry profiling (**Figure 2A**). We were able to robustly detect 3392 proteins in total (**Supplementary Table 1**), from which the matrisome was filtered (in silico defined ECM and related proteins (36)). This led us to identify 151 proteins, further categorized into ECM core (glycoproteins, collagens, and proteoglycans) and associated proteins (ECM-affiliated, ECM regulators, secreted factors). When we compared normalized relative abundance of ECM proteins between CAF and NF, we observed that the abundance of many ECM proteins was significantly increased *(*adjusted p-value < 0.05) in CAF FDMs (**Figure 2A**), compared to NF FDMs, where only a couple of ECM proteins (Srpx2, Ctsl) were significantly decreased. Following this finding, we wanted to further evaluate CAF/NF FDM differences. Here, we validated LC-MS/MS findings (**Figure 2A**) by both immunofluorescence (IF) imaging (**Figure 2B**) and Western Blot analysis (**Figure 2C** and **Supplementary Figure 3**) for selected proteins (periostin, collagen IV, XII, XIV, and lysyl oxidase) in the CAF FDM.

Based on these data, we hypothesized that the ECM produced by CAF versus NF may have altered structure, and we focused on performing label-free second harmonic generation (SHG) imaging (**Figure 2D** and **Supplementary Figure 1A**). Single-plane and maximum intensity projection (MIP) image analysis allowed quantification of ECM density. This showed that CAF FDM possesses denser packed collagen fibers (**Figure 2E**). Using CNA35-mCherry probe, binding fibrillar collagens, we were able to better visualize ECM fibers of the FDMs (**Supplementary Figure 1B**) showing that CAF FDMs have higher intensity of the collagens staining and indeed increased density of fibers (**Supplementary Figures 1C, D**). Analysis of stacks through the z-axis of FDMs concluded that CAF FDM is also significantly thinner than NF FDM’s (**Figure 2F**). Thickness was previously shown to positively correlate with the fibroblasts’ density (43), which in our model is also reflected in the higher density of NFs nuclei in the depositing FDM layer and NFs increased proliferation rate compared to CAFs (**Supplementary Figure 2**). These findings highlighted that CAF FDMs contain more ECM proteins and are thinner as well as denser suggesting, a strong change in CAF FDM mechanics. Therefore, we performed atomic force microscopy (AFM) measurements to determine the stiffness of the FDMs. AFM analysis revealed that CAF FDM stiffness is significantly increased compared to NF FDM (**Figure 2G**). Increased stiffness and density are representative of desmoplastic stroma *in vivo*, therefore, these FDMs present a relevant model for mimicking those differences *in vitro*.

### 3.3 Composition of mammary CAFs FDM reflects desmoplastic changes in pancreatic cancer

**Figure 3:**
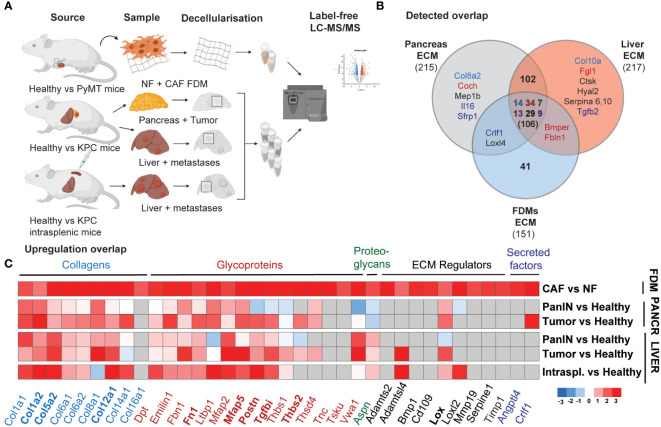
Mammary CAFs deposit compositionally complex matrix reflecting desmoplastic changes in primary and metastatic pancreatic cancer. **(A)** Scheme of the mass spectrometry samples generation for mNF1, mCAF1 FDMs dataset and PDAC dataset, including healthy age-matched tissues and tumors, spontaneous liver metastases from PanIN to PDAC stages and healthy livers age-matched with experimental KPC liver metastases. **(B)** Venn diagram showing an overlap in robustly detected proteins between the datasets. Color-coding is used for depicting matrisome categories (Core matrisome: blue – Collagens, red – Glycoproteins, green – Proteoglycans; Matrisome-associated: dark violet – ECM-affiliated, black – ECM regulators, dark blue – Secreted factors). **(C)** Heatmap of the ECM proteins’ fold changes significantly upregulated (p < 0.05), cutoff logFC >1.59 in mCAF1 vs mNF1 FDMs, compared to the PDAC dataset (tumor conditions vs healthy ones). n=3 samples per group. Grey colored are non-detected proteins. PanIN – pancreatic intraepithelial neoplasia, transition to tumor; Pancreatic tumor presented from the early group. In bold are highlighted genes of the short signature - matched to increasing with progression in the *in vivo* dataset.

Given this outcome, suggesting a strong pro-fibrotic deposition by mammary CAFs, we hypothesized that these changes could represent desmoplastic changes in other cancer types. As PDAC is known to have a highly desmoplastic primary tumor and metastatic site (liver) (34, 44), we generated a proteomic PDAC dataset in order to further explore desmoplastic ECM composition. Here, we utilized our previously published ISDoT (*In Situ* Decellularization of Tissues) method (45) in order to isolate and enrich native ECM proteins from pancreatic and hepatic tissue during pancreatic cancer progression including pancreatic intraepithelial neoplasia (PanIN) and PDAC stages in the KPC (Tg (Pdx1-cre) 6TuvKras^tm4Tyj^Trp53^tm2Tyj^) mouse model. We collected regions from the pancreas of PDAC-developing mice, at PanIN and established tumors stages with early and late formation, as well as from the healthy pancreas of age-matched Pdx-1 Cre mice (**Figure 3A**). The same approach was performed for the liver, where KPC mice developed spontaneous macrometastases. We also included livers, which developed experimental metastases upon intrasplenic injection, where cancer cells drain into the liver through the splenic vein, mimicking the latter stages of metastasis with vast liver macrometastases (**Figure 3A**).

These samples were analyzed by label-free LC-MS/MS, and the abundance of ECM proteins between healthy and tumor conditions at each stage was quantified. Here, we detected 5472 proteins across all conditions, of which 210 were core ECM and ECM-associated proteins (filtered in the same way as the mCAF1/mNF1 proteomic data). Comparison between the different stages of PDAC progression (**Supplementary Figure 4**) allowed identification of 168 significantly upregulated ECM proteins at least in one of the disease stages. These included a number of core matrisome proteins such as collagens (Col1a1, Col1a2, Col5a2, Col8a1, Col12a1) and glycoproteins (Ltbp1, Mfap2, Mfap5), which are upregulated already at the PanIN stage. A few other proteins quantified, such as Postn, Srpx2, Tgfbi, became upregulated during tumor formation, but not at the PanIN stage. We also observed some liver metastasis-specific ECM changes. Vwa1 (Von Willebrand Factor A domain containing 1), for example, was downregulated or unchanged in the pancreas, but significantly upregulated in the liver. As with the CAF/NF proteomics, we also noted an increase in the collagen crosslinking proteins LOX and LOXL2 in tumors and/or metastases. Matching of FDMs dataset with PDAC indicated that most of the proteins were also found *in vivo* (106, all categories) (**Figure 3B**), while among the top list of altered proteins in CAF vs NF most were also upregulated (**Figure 3C**) (cut-off LogFC > 1.59, p-value < 0.05; grey – below detection limit in the *in vivo* dataset). This list of 36 genes we defined as a long ‘desmoplastic signature’ and proteins also gradually upregulated during progression in PDAC, 9 genes, as a short ‘desmoplastic signature’: Col1a2, Col5a2, Col12a1, Fn1, Mfap5, Postn, Tgfbi, Thbs2, Lox.

### 3.4 Desmoplastic signature is enriched in multiple cancer types in human and is prognostic of patient survival

We focused on identifying if the murine ‘desmoplastic signature’ reflects ECM changes in human tumor datasets. The signature genes/proteins were used to define an enrichment score as previously reported (46) from gene expression values of TCGA Pan-Cancer normalized cohort, assessed at both the tumor type and the molecular subtype level.

**Figure 4:**
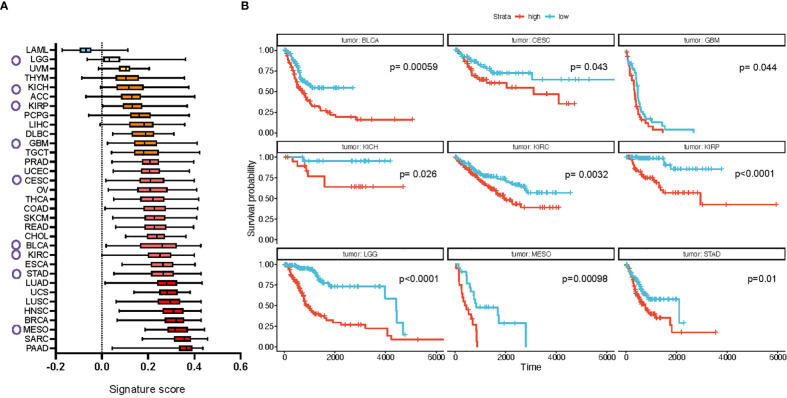
Full CAF FDM signature is enriched in multiple cancer types in human and is prognostic of cancer patients’ survival. **(A)** Difference in the signature expression score (box plots summarize median with min and max). Circles mark cancer types for which high expression of the signature defines poor survival, shown in b (based on TGCA dataset). **(B)** Kaplan-Meier plots of cancer types which show significantly different probability of patients’ survival based on the signature expression. LAML, Acute Myeloid Leukemia; ACC, Adrenocortical carcinoma; BLCA, Bladder Urothelial Carcinoma; LGG, Brain Lower Grade Glioma; BRCA, Breast invasive carcinoma; CESC, Cervical squamous cell carcinoma and endocervical adenocarcinoma; CHOL, Cholangiocarcinoma; COAD, Colon adenocarcinoma; ESCA, Esophageal carcinoma; GBM, Glioblastoma multiforme; HNSC, Head and Neck squamous cell carcinoma; KICH, Kidney Chromophobe; KIRC, Kidney renal clear cell carcinoma; KIRP, Kidney renal papillary cell carcinoma; LIHC, Liver hepatocellular carcinoma; LUAD, Lung adenocarcinoma; LUSC, Lung squamous cell carcinoma; DLBC, Lymphoid Neoplasm Diffuse Large B-cell Lymphoma; MESO, Mesothelioma; OV, Ovarian serous cystadenocarcinoma; PAAD, Pancreatic adenocarcinoma; PCPG, Pheochromocytoma and Paraganglioma; PRAD, Prostate adenocarcinoma; READ, Rectum adenocarcinoma; SARC, Sarcoma; SKCM, Skin Cutaneous Melanoma; STAD, Stomach adenocarcinoma; TGCT, Testicular Germ Cell Tumors; THYM, Thymoma; THCA, Thyroid carcinoma; UCS, Uterine Carcinosarcoma; UCEC, Uterine Corpus Endometrial Carcinoma; UVM, Uveal Melanoma.

Results showed a wide difference in enrichment scores, with tumors from the blood [acute myeloid leukemia (AML)] expressing the lowest scores and metaplastic/squamous tumors [breast (BRCA), head and neck (HNSC), pancreatic (PAAD) cancers and sarcoma (SARC)] the highest (**Figure 4A**). We confirmed these differences at the tumor subtype level, where mesenchymal and immunoreactive subtypes, characterized by large ECM deposition and tissue activation phenomena, topped the landscape of signature enrichment levels. These results are in line with the composition of the signature, that features mostly genes/proteins associated with the ECM, its organization, and TGFβ signaling. The enrichment scores were then divided into quartiles by tumor type and patients in the 1^st^ and 4^th^ quartiles (“low” and “high”, respectively) were compared for overall survival (OS). Results show significant monovariate differences in survival for 9 tumor types (BLCA, CESC, GBM, LGG, KICH, KIRC, KIRP, MESO, STAD (see **Figure 4** legend for abbreviations, all p-values < 0.05; **Figure 4B**), with uveal melanoma (UVM) also being very close to significance (p-value = 0.052) (**Supplementary Figure 5**). We confirmed that a higher level of signature score in these cancer types positively correlates with the abundance of fibroblasts in the samples (**Supplementary Figure 6**). Interestingly, in all cases, a higher signature expression was associated with poorer survival and different tumor types from the same organ or system showed similar results (see, e.g., all kidney neoplasms (KICH, KIRC and KIRP) and bladder cancer (BLCA), lung (LUAD, LUSC, MESO), ovarian and cervical cancers (OVCA and CESC), and high- and low-grade gliomas (LGG and GBM). We performed the same analysis for the short signature which showed a relative increase in expression score for most of the cancer types, prominent for ovarian (OV) and colon (COAD) cancers (**Supplementary Figure 7A**). Survival analysis in addition showed a significant association of the signature expression with poor survival in pancreatic (PAAD) and lung (LUAD) adenocarcinomas (**Supplementary Figure 7B**).

Compared to 100 random signatures from the rest of the matrisome, which is the largest gene “origin” (ontology) in the signature, we observed that both the ‘short’ and ‘long’ signatures obtain much larger scores and much smaller dispersions, strongly suggesting a coordinated and non-random expression of the genes the signatures span across the whole cohort (**Supplementary Figures 9A, B**). This is also reflected in principal component analysis (PCA) overlaid with binned bidimensional probabilities, showing a clear separation between signature and non-signature genes (**Supplementary Figure 9C**). More importantly, we have performed Cox Proportional Hazard (CoxPH) analysis for overall survival (OS), disease-specific survival (DSS) and progression-free interval (PFI) evaluating signature scores together with age, sex and tumor type and found that - in all cases - the signature is an independent estimator of survival, both in the pan-cancer cohort and in the tumors previously identified *via* Kaplan-Meier OS analysis (**Supplementary Figure 10**).

## 4 Discussion

Desmoplastic reactions at both the primary and secondary site in multiple cancers are a hallmark of disease progression (47, 48), and are characterized by an increased deposition of ECM, altered ECM structure and composition as well as changes in the biophysical properties of the surrounding stroma. These alterations are often associated with poor drug response/delivery and poorer clinical outcome (49, 50). However, tools to model and study the effect of the native ECM on cancer progression are still lacking. Here, we utilize FDMs generated by NFs and CAFs, and validate FDM relevance to the *in vivo* situation of desmoplastic cancer. Several studies profiled tumors by single-cell RNA sequencing, showing that CAFs are heterogeneous in both breast and pancreatic tumors (51). Importantly, most of those subtypes are still active producers of the ECM (based on Col1 and Col3). Temporally resolved proteomic studies, which more reliably represent deposited ECM over the course of disease progression, are still very limited (20, 52). Our approach of FDM proteomics can be further applied to fill this gap in exploring ECM deposition by the CAF subtypes.

To generate native ECM in a short timeframe *in vitro*, we used immortalized NFs and CAFs densely seeded on cross-linked gelatin-coated plastic. Removal of fibroblasts results in a layer of the ECM with unique composition, structure, and mechanical properties. Our study reveals that CAF FDM structure is much thinner and denser, mechanically stiffer than NF. These parameters potentially depend on both an increase in the crosslinking enzymes (e.g. LOXL2), packaging collagen fibers core ECM components (FACIT collagens - ColXIV, ColXII), and higher contractility of CAFs (23). CAF FDM top compositional changes match desmoplastic PDAC alterations at the primary and metastatic site and they form a gene signature relevant for identifying desmoplastic state among a broad spectrum of human tumors. Our analysis shows that sarcomas, head and neck, mesothelioma, and lung cancers (ductal carcinoma, adenocarcinoma) also possess pronounced desmoplastic ECM changes at the transcriptional level. For some cancer types, stratification based on our signature is prognostic of the overall survival. Surprisingly, those are also ‘non-desmoplastic’ cancer types with a low signature score (e.g. brain cancers (LGG, GBM) and kidney cancers (KICH, KIRP), suggesting that we potentially lack understanding of which role matrisome plays in their progression. Further experiments, such as evaluation of the non-desmoplastic cancer type cell proliferation on desmoplastic matrices, will shed light on the impact of these stromal changes on either tumor growth, or other parameters driving cancer progression. Interestingly, an earlier study uncovered epigenetic regulation of YAP/TAZ pathway by translocation of JMJD1 histone demethylase in the nucleus on stiffer CAF matrices as a mechanism giving cancer cells a proliferative advantage (53). Other studies so far mainly focused on comparing cancer cell migration on NF versus CAF fibroblast matrices, for instance showing more alignment of the ECM fibers by human prostate and pancreatic CAFs leading to directional migration of cancer cells (54, 55). In this study we did not observe more alignment by CAF indicating tissue or species-specific differences.

We foresee that desmoplastic signature can be a useful tool for identifying patients who could benefit from anti-fibrotic treatment. As some of those targeted treatments were not successful (56), it is critical to consider that pro-fibrotic changes are triggered early on (e.g. in case of PanIN stage in our study), therefore, the stage of disease can be a critical factor for starting the treatment. Further, we need to understand if these ECM components are independent or interdependent in creating desmoplastic response and how preventing their build up can be tuned more effectively, potentially by co-targeting an immune response and CAF heterogeneity. We acknowledge however, that there are limitations when using the TCGA to look at the power of our signature in defining patient outcomes. While different parameters regarding survival are available, there is little information regarding the treatment of these patients and their response, which is a crucial parameter affecting clinical outcome. However, when evaluating signature scores together with age, sex, and tumor type we found that the signature is an independent estimator of survival.

Our datasets also highlight single proteins such as collagen XII, a fibril-associated collagen with interrupted triple helices (FACIT) binding to the surface of collagen fibers and promoting their bundling and compaction. Its presence in the stroma was shown to correlate with epithelial tension mediated by STAT3 signaling in PDAC mouse models (57). Collagen XII was also found to be a prognostic marker of poor patient outcome in colorectal cancer, associated with the myofibroblastic invasive front and liver metastases (58, 59). In breast cancer, its knockdown in CAFs in a cancer cell co-implantation model showed that collagen XII ECM compaction contributes to the metastatic dissemination (52). However, biochemical and structural role of collagen XII, as well as potential therapeutic targeting, remain undefined. This stresses the need for further elucidation of the mechanistic role of ECM components in desmoplasia, and in driving primary tumor progression to metastasis.

## 5 Conclusions

In summary, our study highlights a desmoplastic signature of 36 ECM genes, the expression of which is prognostic of patient survival in 9 cancers. The proteomic datasets presented here can be further explored to investigate the role of specific ECM proteins in cancer progression, and their potential as therapeutic targets. Our study shows that CAFs can be used *in vitro* to generate complex desmoplastic ECM substrates, and that the difference between ‘normal’ and desmoplastic ECM matrices stimulates cancer cell proliferation.

## Data availability statement

The data presented in the study are deposited in the PRIDE repository, accession number: PXD042342.

## Ethics statement

The animal study was reviewed and approved by Danish Inspectorate for Animal Experimentation (permission number #2017-15- 0201-01265).

## Author contributions

Project was conceived by JE, AJ, EH. *In vitro* assays were performed by MR, AJ, EH, FK, DG. Mass spectrometry was performed by MR, ES and analyzed by EH and MR. KPC mice were bred and maintained by MR and LA. *In vivo* work and sample collection were performed by AM-G, SN, KZ. Imaging and FDM image-based characterization was performed by MR. FDM stiffness measurements were performed by LF and HC-S. Western blotting was performed by JS. Signature score and survival analysis in patient datasets were performed by VI. Manuscript was written by AJ, MR, RR, and JE. Project was supervised by RR and JE. All authors contributed to the article and approved the submitted version.
