# Supplementary material for: Fibroblast-derived matrix models desmoplastic properties and forms a prognostic signature in cancer progression
Source: Front Immunol. 2023 Jul 18;14:1154528. doi: 10.3389/fimmu.2023.1154528 (PMC10395327; doi:10.3389/fimmu.2023.1154528)
Supplement: Supplementary file 1 [file DataSheet_1.pdf]

frontiers

Supplementary Material

Fibroblast-derived matrix models desmoplastic properties and forms a prognostic signature in cancer progression

Maria Rafaeva[1#], Adina R.D. Jensen[1#], Edward R. Horton[1#], Kamilla W. Zornhagen[1], Jan E. Strøbech[1], Lutz Fleischhauer[2,3], Alejandro E. Mayorca-Guiliani[1], Sebastian R. Nielsen[1], Dina S. Grønseth[1], Filip Kuś[1], Erwin M. Schoof[1,4,5,6], Luis Arnes[1], Manuel Koch[7,8], Hauke Clausen-Schaumann[2,3], Valerio Izzi[9,10,11], Raphael Reuten[1,12,13*] and Janine T. Erler[1*]

*** Correspondence:** Corresponding Authors: janine.erler@bric.ku.dk; raphael.reuten@pharmakol.uni-freiburg.de

1 Supplementary Figures

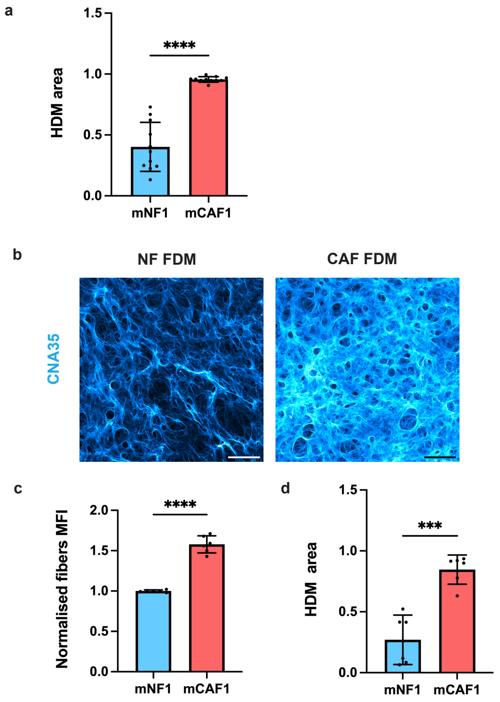

Supplementary Figure 1. a. mNF1 and mCAF1 FDMs density based on SHG maximum intensity projection. Quantified as high density matrix (HDM) area per field of view. n=3 repeats. **b.** Representative images of mNF1 and mCAF1 FDMs imaged after CNA35 probe staining (binding fibrillar collagens). **c.** mNF1 and mCAF1 FDMs ECM fibers mean fluorescent intensity (MFI). n=3 repeats. **d.** mNF1 and mCAF1 FDMs density based on CNA35 staining maximum intensity projection. Quantified as high density matrix (HDM) area per field of view. n=3 repeats. Unpaired t-test, *** – p-value < 0.001, **** – p-value < 0.0001. Scale bars 100 µm.

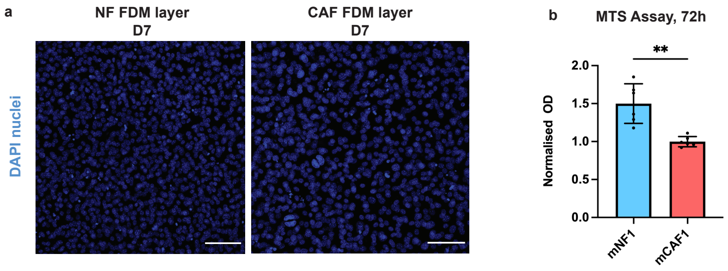

Supplementary Figure 2. mNF1 and mCAF1 confluency in FDM and difference in proliferation. **a.** Representative image of mNF1 and mCAF1 cellular density at day 7 of FDMs deposition. Nuclei stained with DAPI. Scalebar 100 µm. **b.** MTS proliferation assay at day 3 post seeding of mNF1 and mCAF1 in regular culture. n=2, 3 technical repeats per experiment. **– p-value < 0.01.

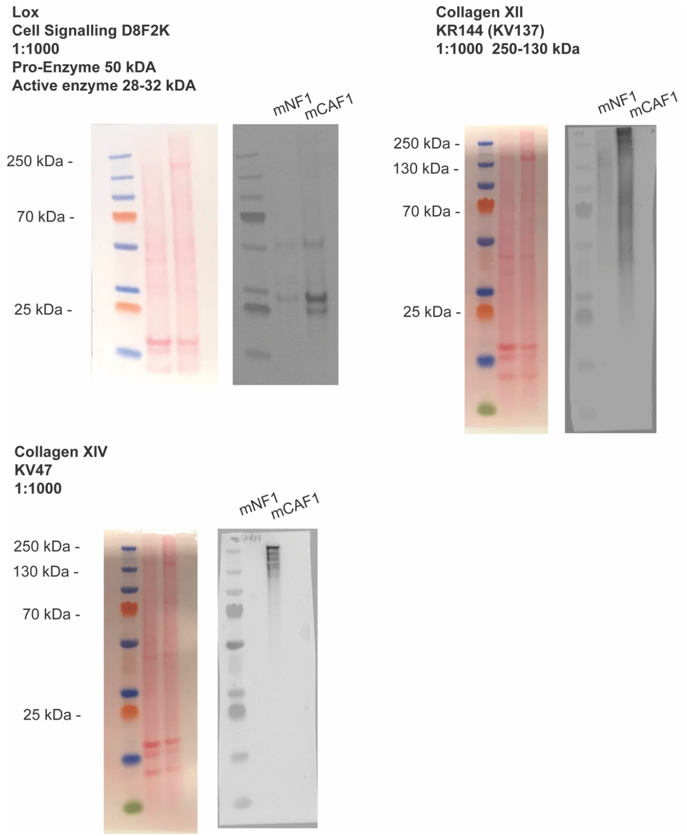

Supplementary Figure 3. *Full membrane scans of the Western blots presented in Figure 2.* with Ponceau dye staining to the left of the scans.

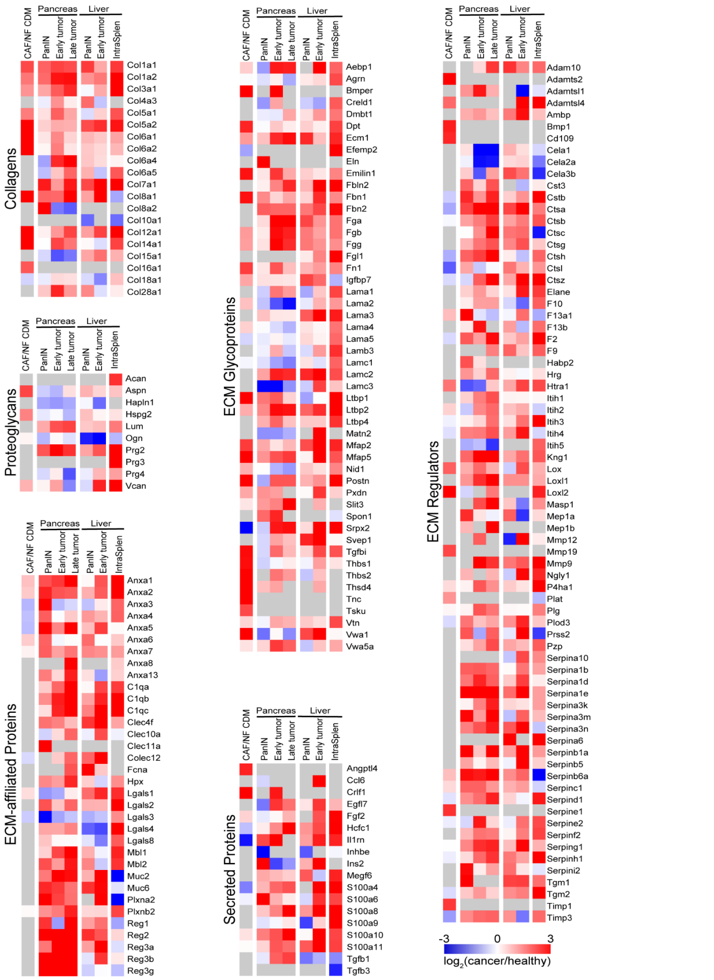

Supplementary Figure 4. *Heatmap of all matrisome changes in PDAC dataset.* Grey values represent non-detected proteins.

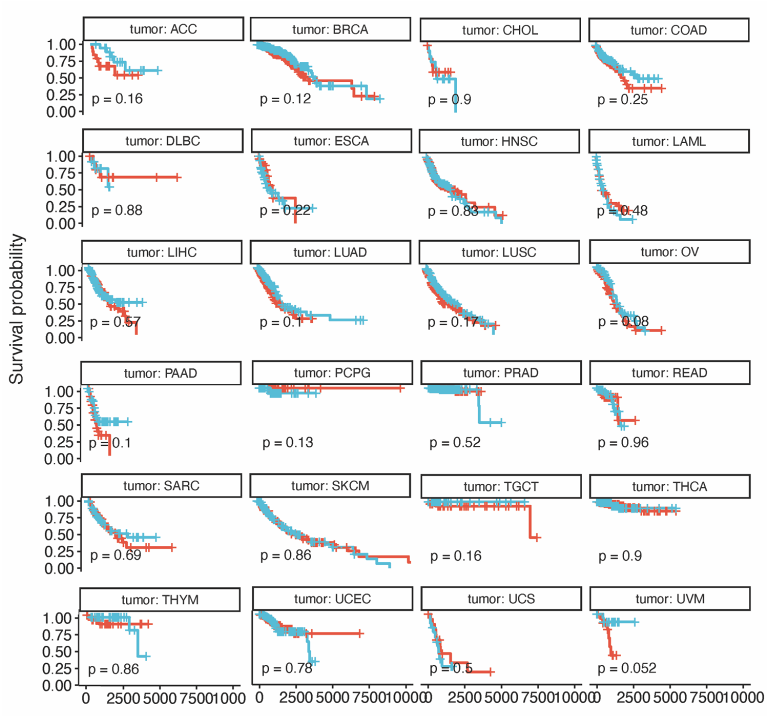

Supplementary Figure 5. *Kaplan-Meir plots for full signature (non-significant p-values).* For abbreviations see Supplementary Fig.4 legend.

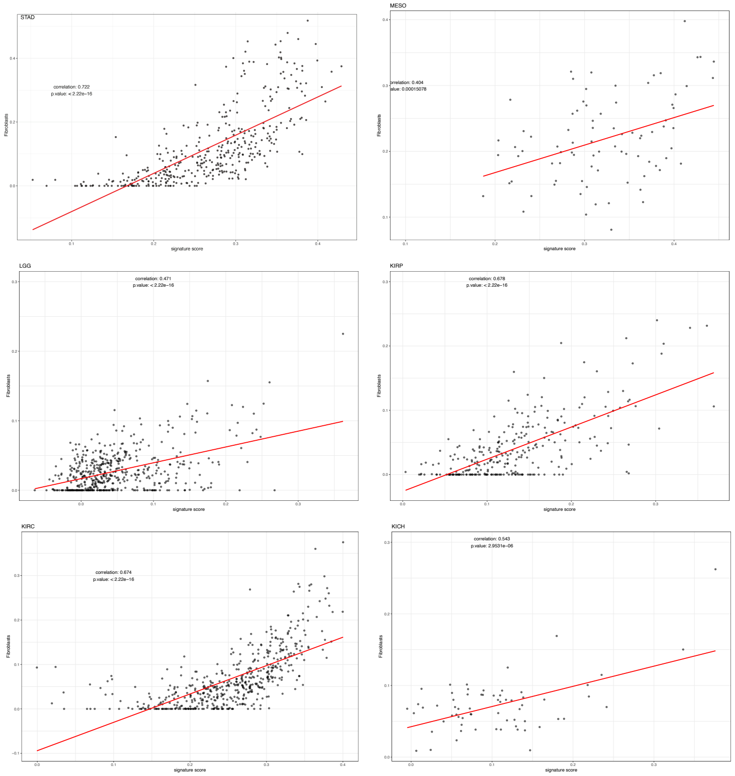

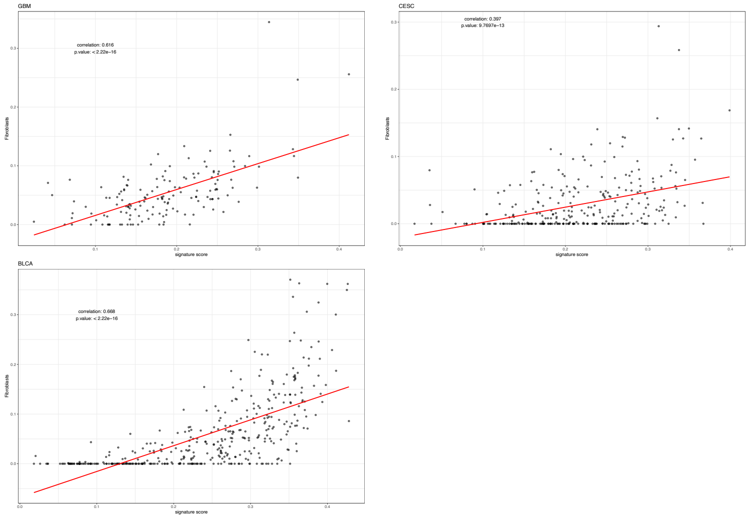

Supplementary Figure 6. *Positive correlation between the signature expression and the relative abundance of fibroblasts within the sample (as obtained from the analysis of the pan-cancer cohort with xCell).*

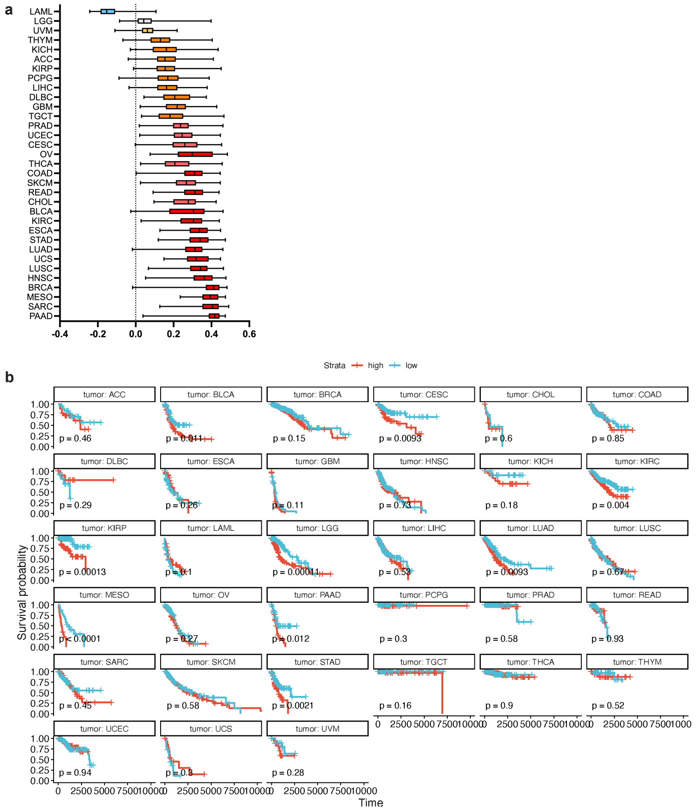

Supplementary Figure 7. *Short CAF FDM signature is enriched in multiple cancer types in human and is prognostic of cancer patients' survival.* **a.** Difference in the signature expression score (box plots summarize median with min and max). **b.** Kaplan-Meier plots of all analysed cancer types. Abbreviations: LAML-Acute Myeloid Leukemia; ACC-Adrenocortical carcinoma; BLCA-Bladder Urothelial Carcinoma; LGG-Brain Lower Grade Glioma; BRCA- Breast invasive carcinoma; CESC-Cervical squamous cell carcinoma and endocervical adenocarcinoma; CHOL-Cholangiocarcinoma; COAD-Colon adenocarcinoma; ESCA-Esophageal carcinoma; GBM- Glioblastoma multiforme; HNSC-Head and Neck squamous cell carcinoma; KICH- Kidney Chromophobe; KIRC-Kidney renal

clear cell carcinoma; KIRP-Kidney renal papillary cell carcinoma; LIHC- Liver hepatocellular carcinoma; LUAD-Lung adenocarcinoma; LUSC-Lung squamous cell carcinoma; DLBC- Lymphoid Neoplasm Diffuse Large B-cell Lymphoma; MESO-Mesothelioma; OV-Ovarian serous cystadenocarcinoma; PAAD-Pancreatic adenocarcinoma; PCPG- Pheochromocytoma and Paraganglioma; PRAD-Prostate adenocarcinoma; READ- Rectum adenocarcinoma; SARC- Sarcoma; SKCM- Skin Cutaneous Melanoma; STAD- Stomach adenocarcinoma; TGCT- Testicular Germ Cell Tumors; THYM- Thymoma; THCA- Thyroid carcinoma; UCS- Uterine Carcinosarcoma; UCEC-Uterine Corpus Endometrial Carcinoma; UVM- Uveal Melanoma.

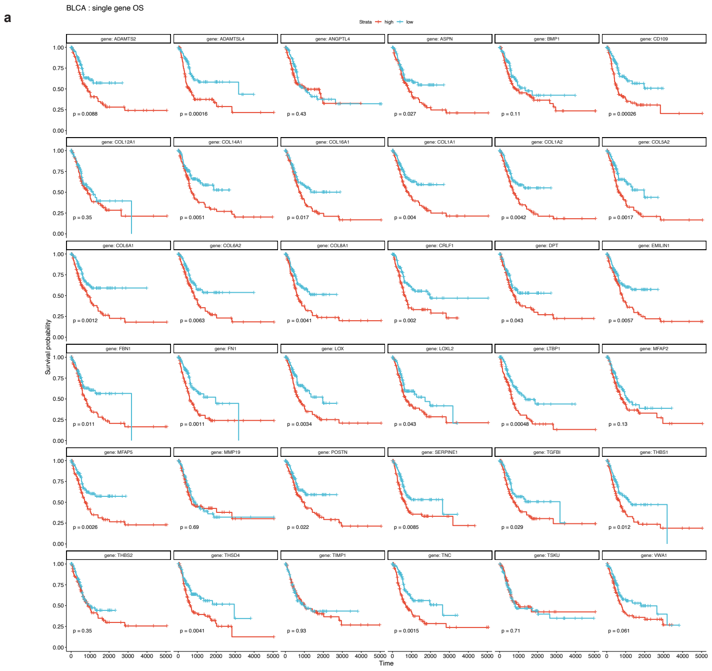

b

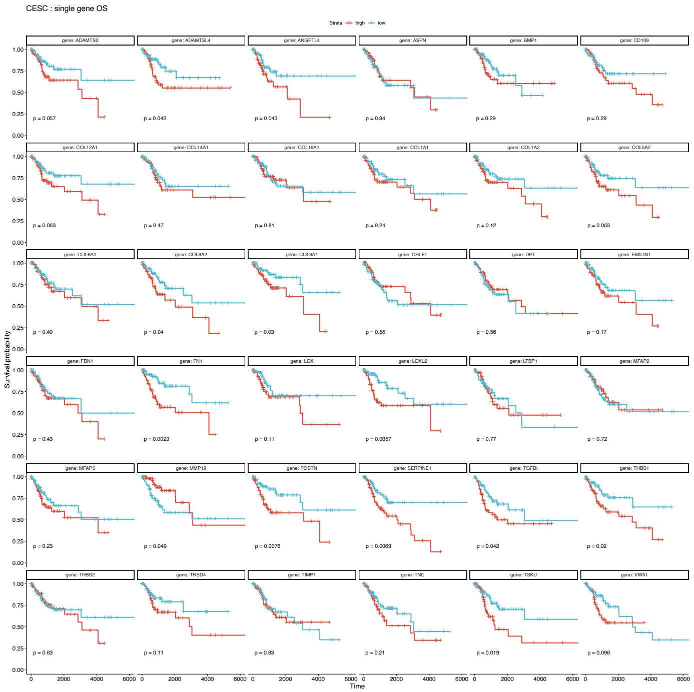
CESC : single gene OS
Strata high low
Survival probability
Time

c

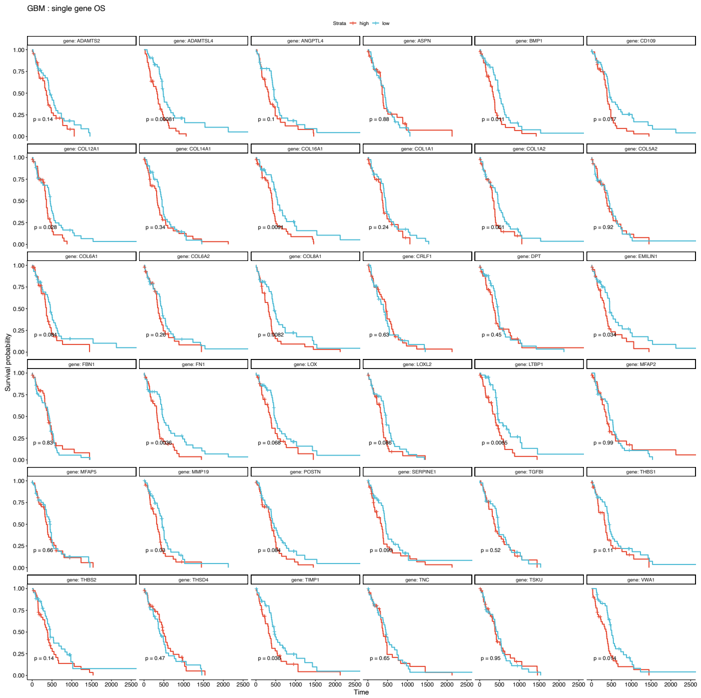
GBM : single gene OS
Strata high low
Survival probability
Time

d

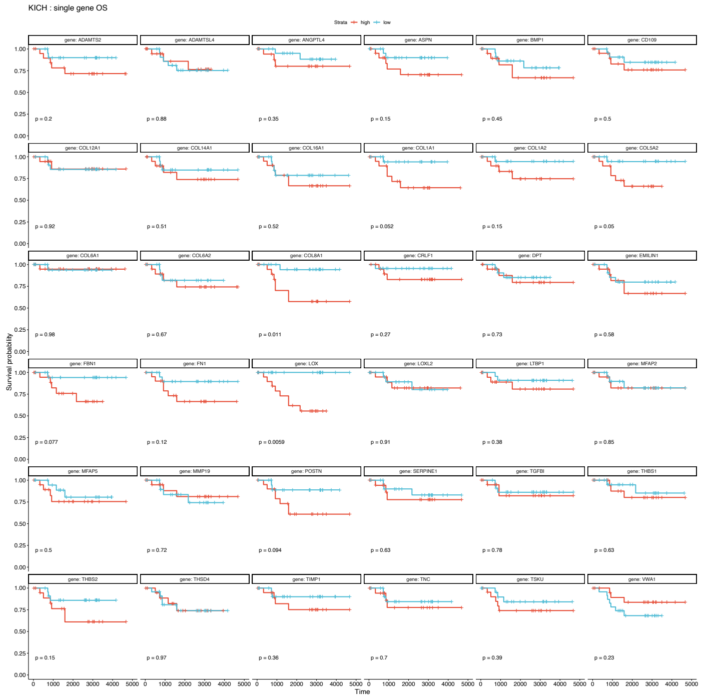
KICH : single gene OS
Strata
high
low
gene: ADAMTS2
p = 0.2
gene: ADAMTSL4
p = 0.88
gene: ANGPTL4
p = 0.35
gene: ASPN
p = 0.15
gene: BMP1
p = 0.45
gene: CD109
p = 0.5
gene: COL12A1
p = 0.92
gene: COL14A1
p = 0.51
gene: COL16A1
p = 0.52
gene: COL1A1
p = 0.052
gene: COL1A2
p = 0.15
gene: COL5A2
p = 0.05
gene: COL6A1
p = 0.98
gene: COL6A2
p = 0.67
gene: COL8A1
p = 0.011
gene: CRLF1
p = 0.27
gene: DPT
p = 0.73
gene: EMILIN1
p = 0.58
gene: FBN1
p = 0.077
gene: FN1
p = 0.12
gene: LOX
p = 0.0059
gene: LOXL2
p = 0.91
gene: LTBP1
p = 0.38
gene: MFAP2
p = 0.85
gene: MFAP5
p = 0.5
gene: MMP19
p = 0.72
gene: POSTN
p = 0.094
gene: SERPINE1
p = 0.63
gene: TGFBI
p = 0.78
gene: THBS1
p = 0.63
gene: THBS2
p = 0.15
gene: THSD4
p = 0.97
gene: TIMP1
p = 0.36
gene: TNC
p = 0.7
gene: TSKU
p = 0.39
gene: VWA1
p = 0.23
Survival probability
1.00
0.75
0.50
0.25
0.00
0
1000
2000
3000
4000
5000
Time

e

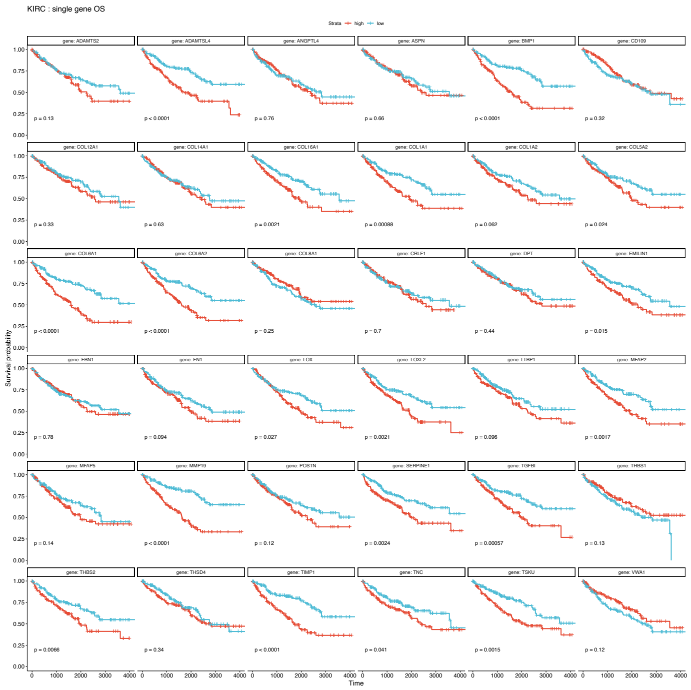
KIRC : single gene OS
Strata
high
low
gene: ADAMTS2
p = 0.13
gene: ADAMTSL4
p < 0.0001
gene: ANGPTL4
p = 0.76
gene: ASPN
p = 0.66
gene: BMP1
p < 0.0001
gene: CD109
p = 0.32
gene: COL12A1
p = 0.33
gene: COL14A1
p = 0.63
gene: COL16A1
p = 0.0021
gene: COL1A1
p = 0.00088
gene: COL1A2
p = 0.062
gene: COL5A2
p = 0.024
gene: COL6A1
p < 0.0001
gene: COL6A2
p < 0.0001
gene: COL8A1
p = 0.25
gene: CRLF1
p = 0.7
gene: DPT
p = 0.44
gene: EMILIN1
p = 0.015
gene: FBN1
p = 0.78
gene: FN1
p = 0.094
gene: LOX
p = 0.027
gene: LOXL2
p = 0.0021
gene: LTBP1
p = 0.096
gene: MFAP2
p = 0.0017
gene: MFAP5
p = 0.14
gene: MMP19
p < 0.0001
gene: POSTN
p = 0.12
gene: SERPINE1
p = 0.0024
gene: TGFBI
p = 0.00057
gene: THBS1
p = 0.13
gene: THBS2
p = 0.0066
gene: THSD4
p = 0.34
gene: TIMP1
p < 0.0001
gene: TNC
p = 0.041
gene: TSKU
p = 0.0015
gene: VWA1
p = 0.12
Survival probability
1.00
0.75
0.50
0.25
0.00
0
1000
2000
3000
4000
Time

f

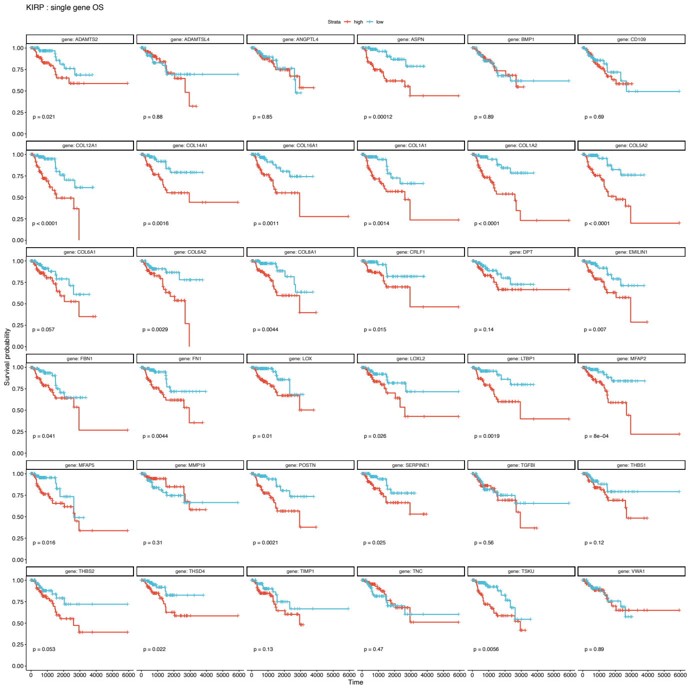
KIRP : single gene OS
Strata high low
Survival probability
Time

g

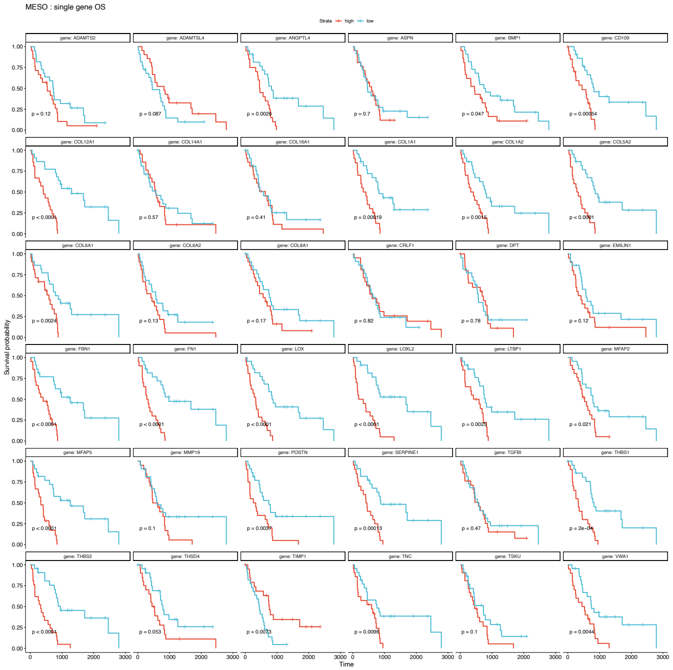
MESO : single gene OS
Strata high low
Survival probability
Time

i

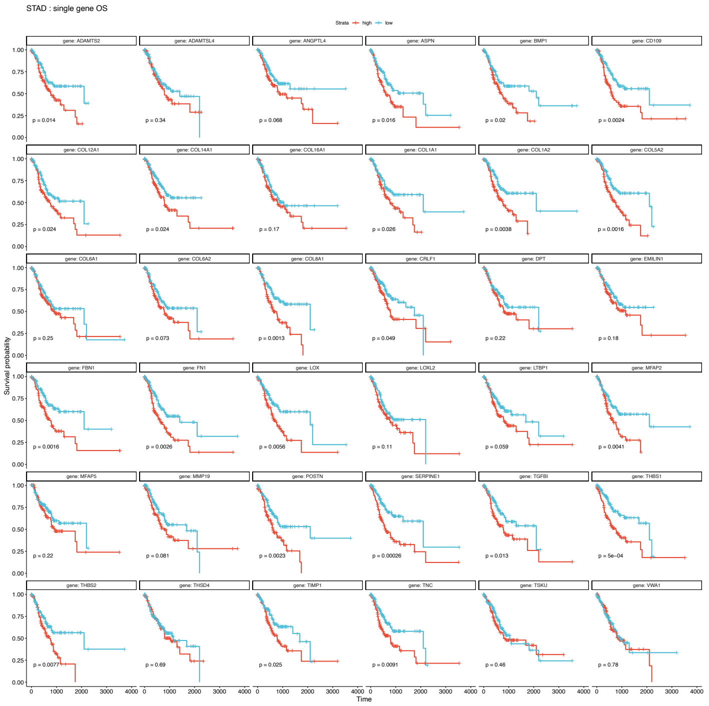

Supplementary Figure 8. *Overall survival based on the high (red) or low (blue) expression of each single gene in the 'long' gene signature, for every cancer type shown in Figure 4.* Indicated on each graph are the p-values. **a**- BLCA; **b**- CESC; **c**- GBM; **d**- KICH; **e**- KIRC; **f**- KIRP; **g**- MESO; **h**- LGG; **i**- STAD cancers.

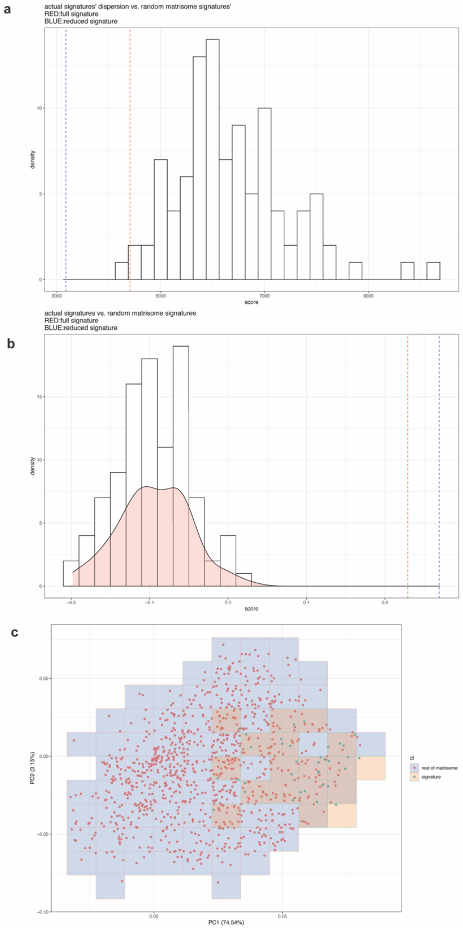

Supplementary Figure 9. *Signature performance comparing to the rest of matrisome.* Comparison of **a)** dispersion and **b)** expression of the short/reduced signature (blue line) and long/full signature (red line) versus 100 random matrisome sets of comparable size. **c)** PCA plot with binned probability hex

superimposed that shows a separation of gene expression between the genes that make the signature and those that do not based on pan-cancer expression profiles.

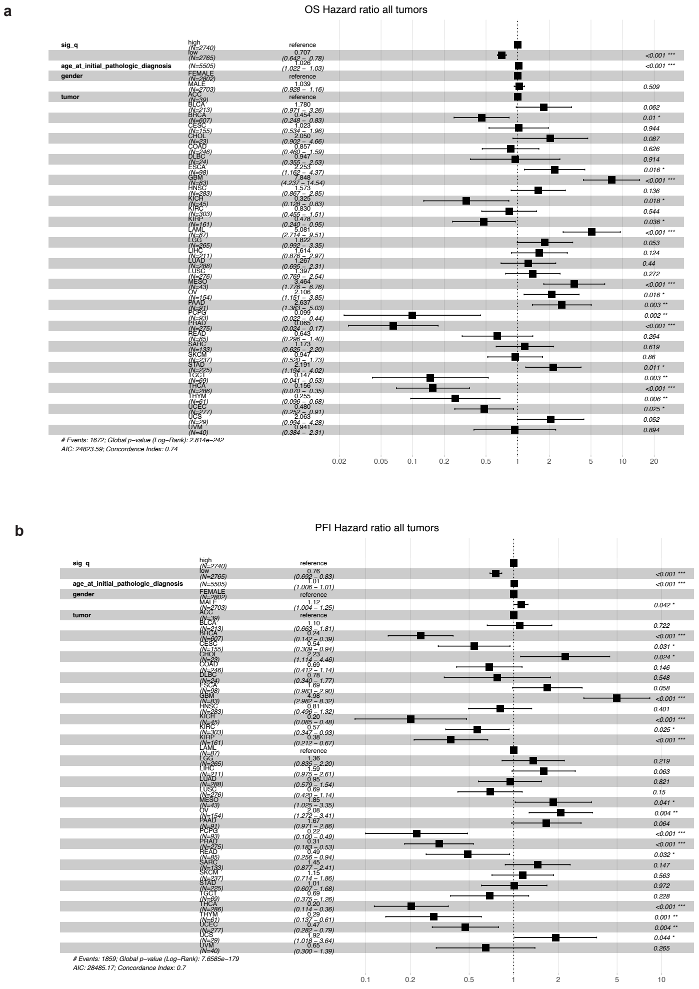

c

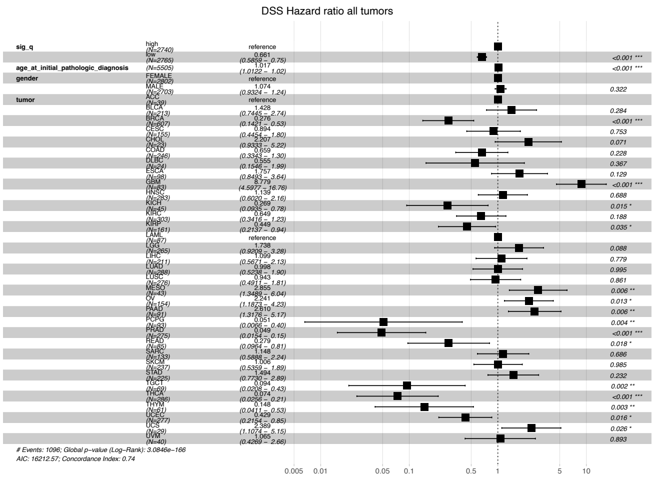

Supplementary Figure 10. *Cox Proportional Hazard (CoxPH) analysis* for **a)** OS (overall survival), **b)** PFI (progression-free survival) and **c)** DSS (disease-specific survival) evaluating signature scores together with age, sex, and tumor type.

Supplementary Tables Legends

Table 1. *Compiled proteomics datasets of NF and CAF FDMs and PDAC.* (1st Tab – NFCAF data matched to PDAC data post-matrisome filtering with the long signature highlighted; 2nd-8th Tabs – Single comparisons data healthy vs cancer including all robustly detected proteins).
